# Which gene did you mean?

**DOI:** 10.1186/1471-2105-6-142

**Published:** 2005-06-07

**Authors:** Barend Mons

**Affiliations:** 1 Biosemantics Group Rotterdam, Department of Medical Informatics, Erasmus MC – University Medical Center Rotterdam, P.O. Box 1738, NL-3000 DR Rotterdam, the Netherlands; 2 MGC – Human and Clinical Genetics, Leiden University Medical Center, Wassenaarseweg 72, 2333AL LEIDEN, the Netherlands

## Abstract

Computational Biology needs computer-readable information records. Increasingly, meta-analysed and pre-digested information is being used in the follow up of high throughput experiments and other investigations that yield massive data sets. Semantic enrichment of plain text is crucial for computer aided analysis. In general people will think about semantic tagging as just another form of text mining, and that term has quite a negative connotation in the minds of some biologists who have been disappointed by classical approaches of text mining. Efforts so far have tried to develop tools and technologies that retrospectively extract the correct information from text, which is usually full of ambiguities. Although remarkable results have been obtained in experimental circumstances, the wide spread use of information mining tools is lagging behind earlier expectations. This commentary proposes to make semantic tagging an integral process to electronic publishing.

## Text mining? ...................Why bury it first and then mine it again?

Recently, Sir Tim Berners-Lee, the inventor of the Web, said: 'Life sciences are particularly suitable for pioneering the Semantic Web. For example, within drug discovery, many databases and information systems used by drug researchers are already in, or are ready to be transformed to, machine-readable formats' [1].

Well, we need our breed of optimists to drive things, and he is obviously one of us.

## Too much to read

Bioinformatics increasingly consists of computer-aided meta-analysis of dispersed articles and database records to assist researchers in the interpretation of massive datasets. Epidemiological studies and high throughput technologies such as microarrays nowadays often lead to sets of potentially relevant papers being identified that surpass human capability for reading, interpretation and synthesis. Aggregating information from many records, followed by logical association of the concepts represented in the full dataset is what I generally refer to here as meta-analysis. Unfortunately, most of the information in the current information sources is not easily accessible, nor, due to ambiguity of the description of concepts in textual format, is it easily readable by computer programs.

## Semantics: a crucial addition to text

If all words had only one possible meaning, computers would be perfectly able to analyse texts. In reality however, words, terms and phrases in text are highly ambiguous. Knowledgeable people have few problems with these ambiguities when they read, because they use context to disambiguate 'on the fly'. Even when fed a lot of semantically sound background information, however, computers currently lag far behind humans in their ability to interpret natural language. Therefore, proper semantic tagging of concepts in texts is crucial to make Computational Biology truly viable. Electronic Publishing has so far only scratched the surface of what is needed.

Open Access publication shows great potential, andis essential for effective information mining, but it will not achieve its full potential if information continues to be buried in plain text. Having true semantic mark up combined with open access for mining is an urgent need to make possible a computational approach to life sciences.

## Databases and pathway tools are not enough

Of course, we have our curated databases, increasingly equipped with fancy analysis and visualisation tools. These databases hold established knowledge on molecules, their interactions and pathways. These databases are definitely useful tools to rapidly get a rough view on 'what may be switched on or involved'. However, the inherent restrictions and downsides of such databases include the great efforts that are needed to keep the error level acceptably low and to keep them up-to-date. Furthermore, biological complexity is more than a composition of multiple, man-imagined pathways. Thus free text continues to be a crucial source of cutting edge information for scientists

A further reason for the continued value of free text is that pre-digested information in databases only contains *explicit knowledge *and will therefore at best cough up 'what somebody already knew', although I as an individual may not be aware of it. Real serendipitous finding of new things and the association between concepts beyond direct co-occurrence is not supported by most existing tools.

## However, text is a nightmare for computers

Unfortunately, free text records are a nightmare of ambiguity. Synonyms and homonyms riddle the records and, in particular, gene and protein names/symbols appear to be impossible to resolve with complete accuracy, based on their textual expression. In an ideal world, scientists would mention formal identifiers from recognized data bases such as EntrezGene or SwissProt in the text, rather than their favourite synonym of the molecule.

With traditional search-based text retrieval tools, this problem was little more than a recurrent nuisance, and current traditional search engine providers probably couldn't care less, as their users are satisfied when they 'find what they want on page one'. But now that computational meta-analysis of textual records is increasingly needed, and the underlying datasets may often consist of tens of thousands of papers, it becomes impossible to manually weed out problems relating to synonyms and homonyms.

## So, like it or not, we have to face the challenge of semantic enrichment

Before any meta-analysis algorithm can be meaningfully applied, semantic analysis and tagging of the underlying texts with unique identifiers for individual concepts is needed. Ideally, this process should be a one-off effort. In a perfect scenario, communication between scientists would take place entirely at the unique concept level. However, this is not reality. What was clear and straightforward in the mind of the researcher after completing the experiments gets lost in a variety of ambiguous expressions during the writing process and ambiguities are produced every minute. Therefore scientific writing can somewhat cynically be called *information burying. *Authors are actually inclined and stimulated to use variable expressions and synonyms, aphorisms and the like to make their article 'readable'. This will not change in the foreseeable future and this means we have to face the challenge of semantic tagging of these texts. To keep the challenge manageable, I will restrain this argument to term identification and not include full analysis of the language structure of sentences as attempted by Natural Language Processing approaches.

## Will authors do it for us?

Scientific text is dry enough as it stands. Should we therefore even contemplate attempts to force authors to make it semantically correct and uniform, according to strict nomenclature or worse, using structured data entry?

I would argue that not only would this approach be completely unfeasible, given the creative and individualistic minds of the producers of scientific knowledge, but in addition, the result of hypothetical success would lead to a totally dull form of scientific literature. In the end, no matter what computational aids we offer, researchers will always keep reading for final evidence. So, we need to make things computer-digestible ***in the background.***

## Will computers do it for us?

With the emerging, improved named entity tagging and semantic tagging technologies, a far more elegant and practical solution is available or just around the corner. Correct (computer) recognition of the concepts denoted by words and phrases in free-text, and the semantic enrichment that comes with it, greatly facilitates direct meta-analysis of subsets of the literature. This pre-processing step also facilitates much more accurate cross-linking of information between articles and databases.

## If yes, why not?

It is surprising that publishers have so far taken almost no steps in this direction, even though it holds almost limitless potential, and it is clear that adding value to plain text is the only perspective publishers may have to charge for textual records in the future. Simple, technically feasible additions to current ontology-based semantic tagging software would allow the development of 'tag as you type' tools that normalise and map ambiguous terms in text to the unique concept they denote and to the corresponding ontologies.

The semantic tagging of new and existing text, notably the well over 14 million papers represented in Medline, seems a daunting perspective at first glance. Researchers are neither educated nor encouraged to take a semantic network approach when publishing their data. As a consequence, even decades after electronic publishing became commonplace, most papers are still ending up as dead, non-interactive pages rusting silently away in electronic archives until a search engine happens to be fed with the correct combination of keywords to retrieve them.

## Or...will computers and authors do it together?

**Figure 1:**
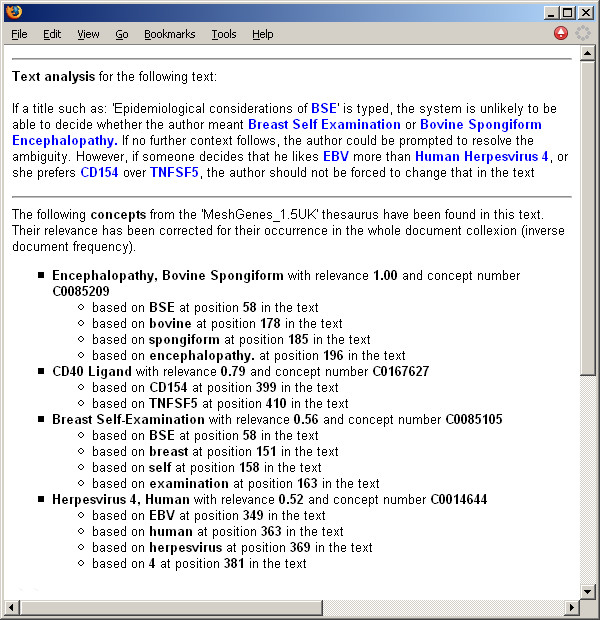
The part of text of this editorial that was marked in italics was fed to an existing semantic tagging tool and it can be seen in the figure how different expressions are mapped to the same concept number.

In my opinion, author-interactive publication tools should ***not ***force the writing scientist to use specific terms from pre-structured lists and nomenclatures. Trying to do exactly that, is what made most efforts of nomenclature standardization ineffective. In contrast, the very valuable efforts of the HGNC's, Entrezgenes and SwissProts of this world should be used to *disambiguate terms on the fly *and only ask the author for assistance in the rare cases the ontology driven system can not make an informed decision about the meaning of a term, for example in case a homonym is used without sufficiently distinguishing context surrounding it. If a title such as: 'Epidemiological considerations of BSE' is typed, the system is unlikely to be able to decide whether the author meant Breast Self Examination or Bovine Spongiform Encephalopathy. If no further context follows, the author could be prompted to resolve the ambiguity. However, if someone decides that he likes EBV more than Human Herpesvirus 4, or she prefers CD154 over TNFSF5, the author should not be forced to change that in the text, as long as the semantic tag added to that term in the background is linked to the unique concept identifier of the virus or the gene in the leading ontologies and nomenclature data bases (see figure 1). Obviously, some extra work will be asked from the author, but not anywhere near as much as most critics of the semantic web idea seem to expect.

In general, the expectation would be that the vast majority of relevant concepts in biomedical text could be correctly tagged on the fly and in the background without bothering the author at all, other than to resolve the occasional ambiguity, and to review the overall markup once complete.

## Yes, but..

Obviously, the critics of the Semantic Web approach will bring in their arguments about the difficulties with tagging the legacy documents and the issue of the rapidly developing knowledge, reflecting in ontology changes. Obviously they have a potential point: If only recently published documents are being tagged, computational tools that rely on the tagging will be very restricted in their resources. The technological approach proposed here will enable us to tag existing texts on the fly with high accuracy, by taking care of the 'easy tagging' and interact with the author or the reader only to resolve the few remaining ambiguities. Moreover, as the tagging is not necessarily static, updated ontologies will lead to (optional) updated proposed tags each time the text is retrieved. Tagging would thus effectively be limited to texts that are still used. If an article is never retrieved by anyone it is probably of limited urgency to tag it.

## So, let's do it!

Given the enormous impact a collective semantic enrichment effort would make, particularly on many aspects of bioinformatics research and computational biology, this process should have started many years ago. The Open Access publishers, but *BMC Bioinformatics *in particular, should take the lead and promote the use of semantic tagging, probably starting at one of the time points at which stubborn scientists like me are most likely to accept any guidance, and that is when they submit a manuscript.

## References

[B1] http://www.thestandard.com/internetnews/001301.php.

